# Insecticidal activity of fluralaner (Exzolt^®^) administered to *Gallus gallus domesticus* against triatomines (Hemiptera, Reduviidae, Triatominae)

**DOI:** 10.1186/s13071-024-06276-8

**Published:** 2024-05-08

**Authors:** Luanderson Cardoso Pereira, Nathalie de Sena Pereira, Andressa Noronha Barbosa da Silva, Clarice de Freitas Bezerra, Kivia Millana de Sousa, João Ciro Fagundes Neto, George Harisson Felinto Sampaio, Carlos Ramon do Nascimento Brito, Rita de Cássia Moreira Souza, Lúcia Maria da Cunha Galvão, Antônia Claudia Jácome da Câmara, Manuela Sales Lima Nascimento, Paulo Marcos Matta Guedes

**Affiliations:** 1 Graduate Program in Parasitary Biology, Federal University of Rio Grande do Norte, Natal, Brazil; 2 Graduate Program in Biological and Health Sciences, Federal University of Vale do São Francisco, Petrolina, Brazil; 3 Graduate Program in Pharmaceutical Sciences, Federal University of Rio Grande do Norte, Natal, Brazil; 4 Department of Microbiology and Parasitology, Federal University of Rio Grande do Norte, Natal, Brazil; 5 Zoonosis Surveillance Unit at Natal, Rio Grande do Norte, Natal, Brazil; 6 Graduate Program in Health Sciences, Federal University of Rio Grande do Norte, Natal, Brazil; 7 Department of Clinical and Toxicological Analyses, Federal University of Rio Grande do Norte, Natal, Brazil; 8 Instituto René-Rachou—FIOCRUZ, Belo Horizonte, Minas Gerais Brazil

**Keywords:** Chagas disease, Fluralaner, Exzolt^®^, Systemic insecticide, *Triatoma*, *Rhodnius*, Chicken

## Abstract

**Background:**

*Triatoma infestans, Triatoma brasiliensis*, *Triatoma pseudomaculata* and *Rhodnius prolixus* are vectors of *Trypanosoma cruzi*, the etiological agent of Chagas disease. Chickens serve as an important blood food source for triatomines. This study aimed to assess the insecticidal activity of fluralaner (Exzolt^®^) administered to chickens against triatomines (*R. prolixus, T. infestans, T. brasiliensis* and *T. pseudomaculata*).

**Methods:**

Twelve non-breed chickens (*Gallus gallus domesticus*) were randomized based on weight into three groups: negative control (*n* = 4); a single dose of 0.5 mg/kg fluralaner (Exzolt^®^) (*n* = 4); two doses of 0.5 mg/kg fluralaner (Exzolt^®^) (*n* = 4). Nymphs of 3rd, 4th and 5th instars of *R. prolixus, T. infestans, T. brasiliensis* and *T. pseudomaculata* (all *n* = 10) were allowed to feed on chickens before treatment, and at intervals of 1, 7, 14, 21, 28, 35 and 56 days after treatment, with insect mortality determined.

**Results:**

Treatment with two doses of fluralaner showed higher insecticidal efficacy against *R. prolixus, T. infestans* and *T. brasiliensis* compared to the single-dose treatment. Similar insecticidal efficacy was observed for *T. pseudomaculata* for one and two doses of fluralaner. Insecticidal activity of fluralaner (Exzolt^®^) against triatomine bugs was noted up to 21 and 28 days after treatment with one and two doses of fluralaner, respectively.

**Conclusions:**

The results demonstrate that treatment of chickens with fluralaner (Exzolt^®^) induces insecticidal activity against triatomines for up to 28 days post-treatment, suggesting its potential use as a control strategy for Chagas disease in endemic areas.

**Graphical Abstract:**

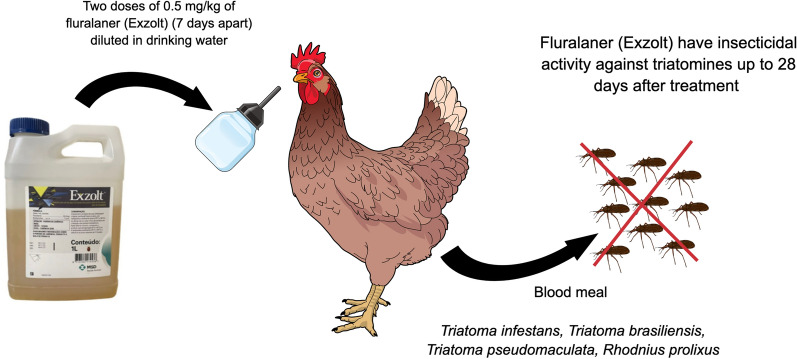

## Background

*Trypanosoma cruzi* (Chagas, 1909) (Kinetoplastida, Trypanosomatidae) is the etiologic agent of Chagas disease. Approximately 6–7 million people are currently infected with this parasitic infection and 75 million live in areas where they are at risk of contracting it [1]. The main type of *T. cruzi* transmission in endemic areas of Latin America is vectorial [1]. Triatomines (Hemiptera: Reduviidae: Triatominae), which are insects, are responsible for the vector-borne transmission of *T. cruzi* [2]*. Triatoma infestans* (Klug, 1834), *Triatoma dimidiata* (Latreille, 1811), *Triatoma brasiliensis* (Neiva, 1911), *Rhodnius prolixus* (Stal, 1859) and *Panstrongylus megistus* (Burmeister, 1835) are considered the most important vectors of the parasite in Latin America [3–5]. *Triatoma infestans* is the main vector in Southern Cone countries, such as Bolivia, Argentina, Paraguay and Chile [3, 6]. *Triatoma dimidiata* has epidemiological importance in the transmission of the parasite in northern South America (Ecuador, Colombia, Venezuela and Peru), Central America (Costa Rica, Honduras, El Salvador, Guatemala, Nicaragua, Panama and Belize) and Mexico [3]. *Rhodnius prolixus* is an important vector in Venezuela, Colombia and Central America [7] and *P. megistus* in Brazil, Bolivia, Paraguay, Uruguay and Argentina [3, 8]. *Triatoma brasiliensis* is the main vector of the parasite in Northeast Brazil, while *T. pseudomaculata* (Corrêa & Espínola, 1964) holds secondary epidemiological significance in this area [9–12].

The control of Chagas disease is mainly based on entomological surveillance in endemic areas and spraying of pyrethroid insecticide with residual action in domestic and peridomestic environments [5, 13]. However, the residual effect of the insecticide only remains for a few weeks (4–12 weeks) in the peridomestic environment due to conditions such as wind, rain and sunlight [14, 15]. Furthermore, triatomines can remain in places where the sprayed insecticide cannot reach, such as within piles of tiles and wood. Therefore, epidemiological surveillance and reapplication of insecticides are often necessary to prevent environmental recolonization. Epidemiological surveillance and insecticide reapplication are costly measures and require the mobilization of a large number of people and amount of equipment [5]. Furthermore, the use of insecticides has stimulated the emergence of populations of pyrethroid-resistant *T. infestans* [16–19] and *R. prolixus* [19, 20]. Indeed, the implementation of new strategies for triatomine control, in conjunction with existing methods, is important to diminish both insect vector populations and human infection rates.

In the peridomestic environment, some ecotopes are more favorable for the development of triatomines [21], with chicken coops being among the most heavily infested [9, 22–25]. The distribution of triatomines is closely linked to a food source, and chicken coops, providing both food and shelter, facilitate the reproduction and development of these insects. Chickens kept in coops can therefore play a crucial role in the maintenance of insect colonies near human dwellings, and thus increase the risk of *T. cruzi* transmission to humans [26–30]. Thus, chickens are promising targets for interventions, as their treatment can reduce the triatomine population and prevent human infection with *T. cruzi*.

Fluralaner (Exzolt^®^) belongs to a novel class of systemic insecticidal drugs called isoxazolines. These drugs act on chloride channels coupled to gamma-aminobutyric acid and channels coupled to L-glutamate, exhibiting a high selectivity for insect neurons over mammalian neurons. Fluralaner (Exzolt^®^) is administered to chickens through their drinking water and demonstrates potent acaricidal activity against red spider mites (*Dermanyssus gallinae*) and northern spider mites (*Ornithonyssus sylviarum*) [31–33]. The administration of fluralaner (Bravecto^®^) to dogs generates insecticidal activity against triatomines such as *T. infestans* [34, 35], *T. brasiliensis* [36] and *R. prolixus* [37]. A recent study demonstrated that administering fluralaner (Bravecto^®^, formulation for dogs) to chickens resulted in insecticidal activity against triatomine bugs [*Triatoma gerstaeckeri* (Stal, 1859)] for up to 15 days after treatment [38]. Hence, the objective of this study was to assess the insecticidal activity of fluralaner (Exzolt^®^) administered to chickens against triatomines (*R. prolixus*, *T. brasiliensis*, *T. infestans* and *T. pseudomaculata*) that are epidemiologically significant in the transmission of *T. cruzi* to humans in Latin America, an area endemic for Chagas disease.

## Methods

### Insects

The specimens of *T. infestans, T. brasiliensis, T. pseudomaculata* and *R. prolixus* used in the experiments, totaling 960 nymphs of the 3rd, 4th and 5th instars of each species, were obtained from a colony established at the Laboratory of Immunoparasitology and Laboratory of Biology of Chagas disease at UFRN. Colonies of *T. brasiliensis* and *T. pseudomaculata* originated from insects captured in the cities of Caraúbas and Serra Negra do Norte, state of Rio Grande do Norte, Brazil. *Triatoma infestans* (from Espinosa, Minas Gerais, Brazil) and *R. prolixus* (from Honduras) were acquired from colonies of the Instituto René Rachou (FIOCRUZ-MG, Brazil). Insect colonies are maintained through weekly feeding on Swiss mice. Triatomines are kept in glass cages which have screening at the bottom, and are maintained in an insectary under controlled conditions of darkness, 50% humidity and a temperature of 28 °C. For the experiments, each chicken was exposed to ten nymphs of the 3rd, 4th or 5th instars of the different species of triatomines on each trial day. Different nymph instars (3rd, 4th and 5th) were randomly distributed in the containers. Three experimental groups with four chickens per group were evaluated, totaling 960 specimens for each species used (10 nymph × 8 time points × 3 different experimental groups × 4 chickens = 960). The nymphs of *T. infestans, T. brasiliensis, T. pseudomaculata* and *R. prolixus* were deprived of food for 30 days and then placed in a 50-mL plastic pots with a screen lid. Triatomines used in the experiments were not infected with *T. cruzi.*

### Chickens

Twelve non-breed chickens (*Gallus gallus domesticus*), commonly called “capoeira chicken” or “caipira chicken,” aged between 1 and 3 years, were selected and housed at a private poultry farm in Parnamirim, Rio Grande do Norte, Brazil. The birds were kept in cages, with each cage containing four chickens. They were provided with a commercial laying chow (Fazendinha Poedeira, Dourado Rações, Brazil) and had access to water ad libitum. It is noteworthy that neither the chickens nor their environment were treated with insecticide for the 12 months preceding the study, or throughout the study’s duration.

### Drug

Chickens were treated with fluralaner (10 mg/ml) (Exzolt^®^, Merck Animal Health, USA), which was added to their drinking water for a period of 24 h. Fluralaner (Exzolt^®^) was administered using two therapeutic schemes: a single dose of 0.5 mg/kg; two doses of 0.5 mg/kg, with a 7-day interval between the doses. The required volume of Exzolt® was calculated based on the body weight of the birds. The volume of water consumed by the chickens was estimated the day before the administration of Exzolt®, and the volume required before medication was determined by the average weight of the treated birds. On the day of each administration, medicated water was prepared and provided to the birds for 24 h. There was no other source of drinking water available during the medication period.

### Study design

**Fig. 1:**
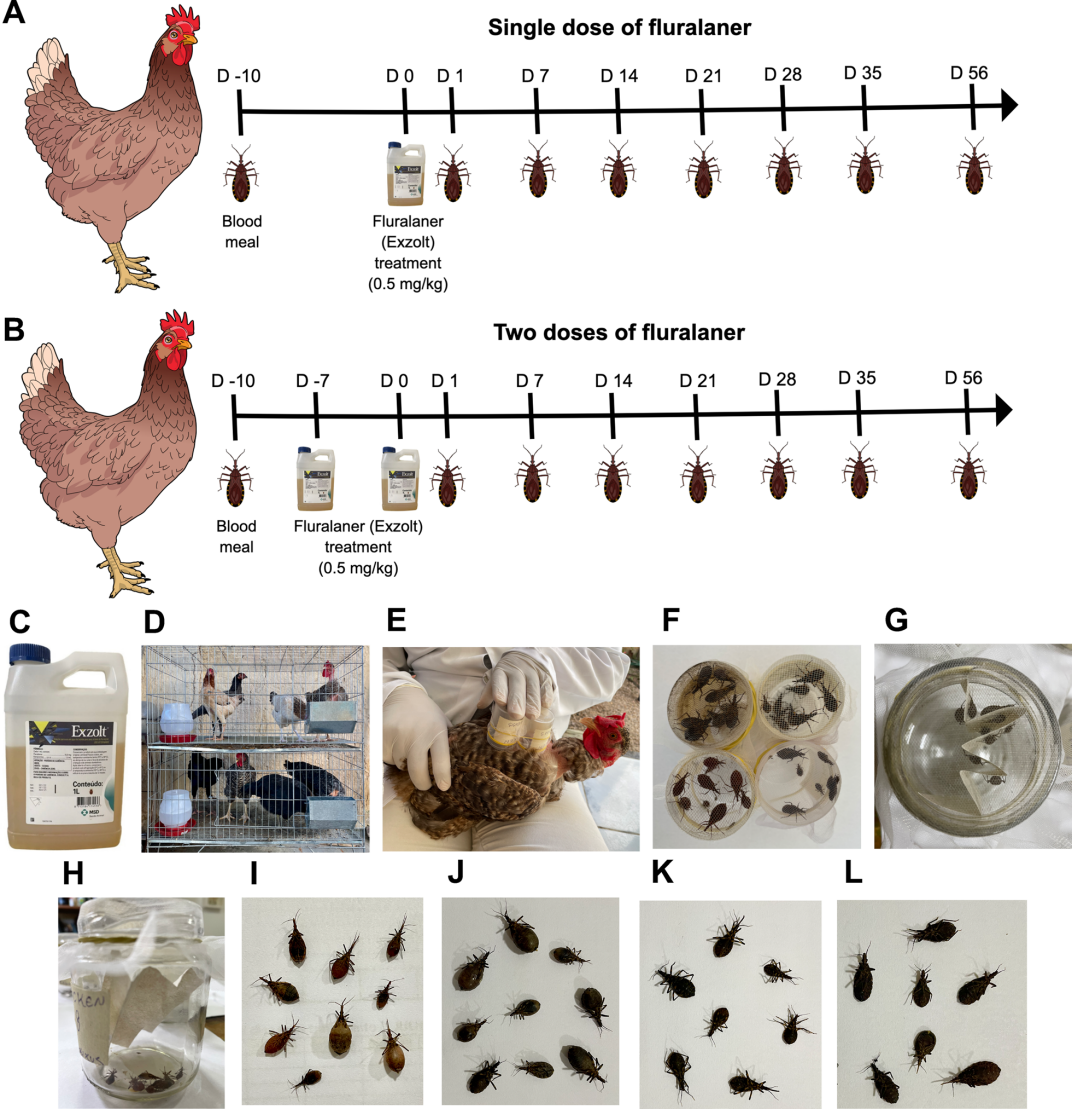
**A–K** Bioassay: chickens treated with fluralaner (Exzolt^®^) and subjected to a blood meal taken by triatomines to determine insecticidal activity. Chickens were treated with one (**A**) or two (**B**) 0.5 mg/kg doses of fluralaner (Exzolt^®^), which was added to their drinking water (**C, D**) for 24 h. Insecticidal activity was determined at intervals of 1, 7, 14, 21, 28, 35 and 56 days after treatment (**A**,** B**). Chickens were constrained by a researcher, and 50-ml plastic pots covered with a fine mesh containing nymphs (*n* = 10) of the 3rd, 4th and 5th stages of each triatomine species (*Rhodnius prolixus, Triatoma infestans, Triatoma brasiliensis* and *Triatoma pseudomaculata*) were positioned and maintained for 30 min on the ventral region of the chickens for the insects to take a blood meal (**E**). After the blood meal (**F**), the insects that had not fed were removed and the engorged insects were transferred to a 300-ml glass container (**G**). The insects were placed in an insectarium (in the dark, at 50% humidity and 28 °C) and observed for 7 days to determine triatomine mortality (**H**). Engorged nymphs of the 3rd, 4th, and 5th instars of *R. prolixus* (*n* = 9) (**I**), *T. infestans* (*n* = 9) (**J**), *T. brasiliensis* (*n* = 7) (**K**) and *T. pseudomaculata* (*n* = 7) (**L**) that died after their blood meal from chickens treated with two doses of 0.5 mg/kg fluralaner

Twelve non-breed chickens (*G. gallus domesticus*) were randomly assigned based on weight to three groups: untreated (control) (*n* = 4); treated with a single dose of 0.5 mg/kg fluralaner/Exzolt^®^ (*n* = 4); treated with two doses of 0.5 mg/kg fluralaner/Exzolt^®^ (*n* = 4). The purpose of this was to assess insecticidal activity against triatomines. Nymphs of the 3rd, 4th and 5th instars of *T. infestans, T. brasiliensis, T. pseudomaculata* and *R. prolixus* (*n* = 10) were allowed to feed on chickens before treatment, and at intervals of 1, 7, 14, 21, 28, 35 and 56 days after treatment, with insect mortality determined. The chickens were placed in the dorsal decubitus position by a researcher, with their feet restrained with bandages to minimize movement, to allow the insects to take a blood meal. Containers with a fine mesh lid, each containing ten nymphs of each triatomine species, were positioned on the ventral region of the birds, and the insects allowed to feed for 30 min (Fig. 1). Following the blood meal, the feeding efficiency of the triatomine nymphs was semiquantitatively assessed, to categorize them as fully engorged, partially engorged, slightly engorged or not fed, in accordance with Reithinger [39]. Then, the insects were placed in an insectarium (28 °C, 50% humidity, in darkness) in the Immunoparasitology Laboratory at UFRN. The insects were monitored daily for a period of 7 days to determine mortality.

### Statistical analysis

Insecticidal efficacy of fluralaner (Exzolt^®^) treatment against different triatomine species was assessed based on the average percentage mortality observed among the insects that fed on treated chickens, as no mortality was observed among insects that fed on untreated control chickens. To evaluate the insecticidal activity of fluralaner/Exzolt^®^ treatment against triatomines, differences in insect mortality were determined using a generalized linear mixed model (GLMM) for repeated measures. The model incorporated fixed effects such as fluralaner treatment, feeding efficiency and engorgement level of insect, as well as time, and the interaction between these variables. The AR(1) covariance matrix was employed in the statistical model. Pairwise comparisons were conducted using the Bonferroni test. The analyses were performed using SPSS 20.0 (SPSS, Chicago, IL), and differences were considered statistically significant when *P* < 0.05. Graphs were made using PRISM 9.0 software (GraphPad, San Diego, CA).

## Results

### Treatment of chickens with fluralaner (Exzolt^®^) does not affect the feeding rate of triatomines

**Fig. 2:**
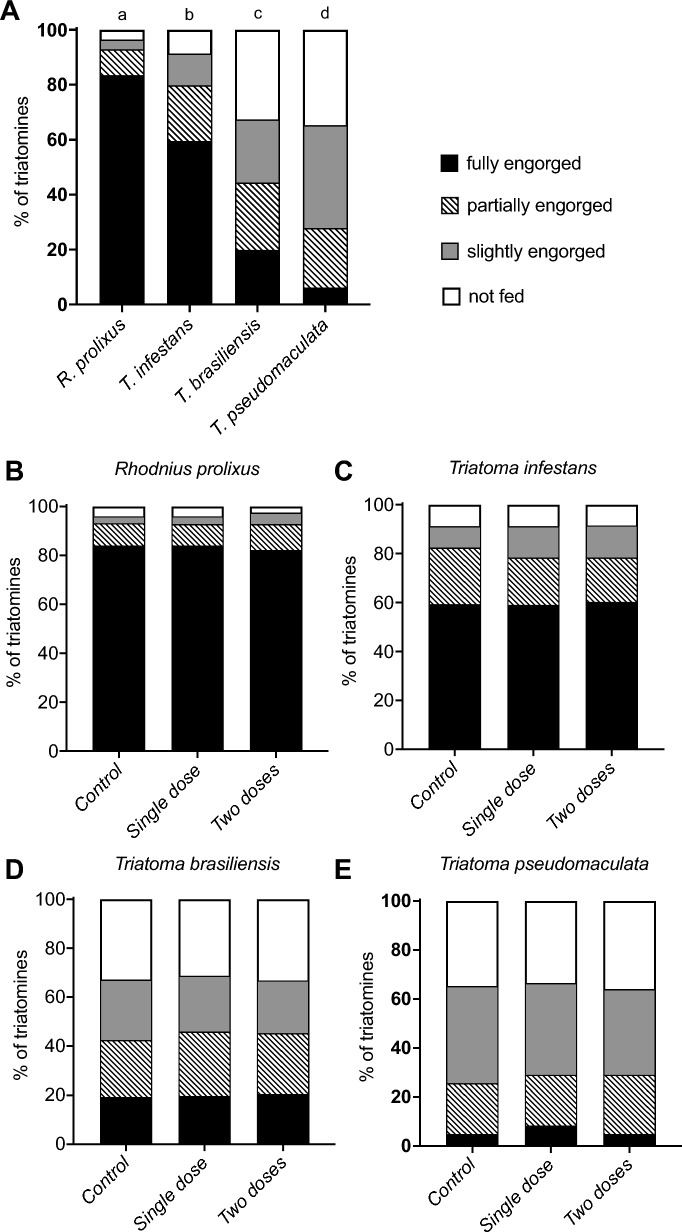
**A–E** Fluralaner (Exzolt^®^) treatment of chickens does not impact the feeding rate of triatomines. Feeding success (**A**) and engorgement level of *Rhodnius prolixus* (**B**), *Triatoma infestans* (**C**), *Triatoma brasiliensis* (**D**) and *Triatoma pseudomaculata* (**E**) after feeding on untreated control chickens and chickens treated with a single dose of 0.5 mg/kg fluralaner or two doses of 0.5 mg/kg fluralaner. Triatomine engorgement level was semiquantitatively determined before treatment and at 1, 7, 14, 21, 28, 35 and 56 days after treatment, whereby the insects were categorized as fully engorged, partially engorged, slightly engorged or not fed. Bars with different lowercase letters indicate statistically significant difference at *P* < 0.05

**Fig. 3:**
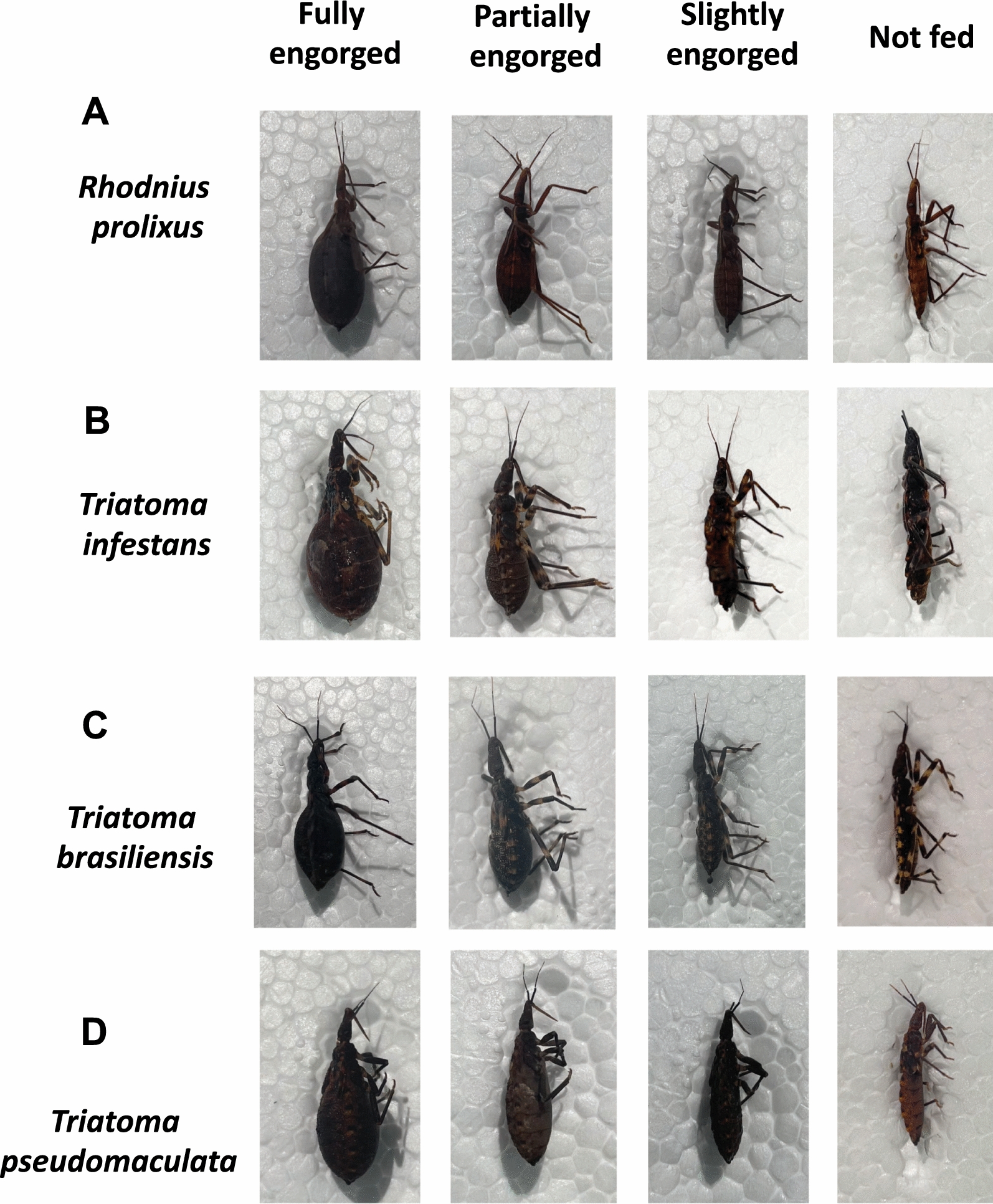
**A–D** Engorgement level of triatomines after a blood meal on chickens. *Rhodnius prolixus* (**A**), *Triatoma infestans* (**B**), *Triatoma brasiliensis* (**C**) and *Triatoma pseudomaculata* (**D**) engorgement levels ware semiquantitatively determined, whereby they were classified as fully engorged, partially engorged, slightly engorged or not fed, after a blood meal on untreated control chickens and chickens treated with a single dose of 0.5 mg/kg fluralaner or two doses of 0.5 mg/kg fluralaner. All images are of 5th instar nymphs

General feeding success on chickens for *R. prolixus* (*n* = 960),* T. infestans* (*n* = 960),* T. brasiliensis* (*n* = 960) and *T. pseudomaculata* (*n* = 960), including all groups of birds, was 96.4%, 91.3%, 67.5% and 65.3%, respectively (Fig. 2A). Specimens of *R. prolixus*, *T. infestans, T. brasiliensis* and *T. pseudomaculata* were 83.4%, 59.8%, 19.9% and 6.1% fully engorged, respectively; 9.4%, 20.2%, 24.5% and 21.7% partially engorged, respectively; 3.5%, 11.5%, 23.0% and 37.4% slightly engorged, respectively; 3.5%, 8.6%, 32.5% and 34.6% not fed, respectively (Figs. 2A, 3). Taken together, the results suggest a higher feeding success rate for *R. prolixus*, followed by *T. infestans, T. brasiliensis* and *T. pseudomaculata*.

There was no difference (*P* = 1.000) in feeding success and engorgement level between triatomines of the same species that fed on untreated control chickens and those treated with a single dose of 0.5 mg/kg fluralaner or with two doses of 0.5 mg/kg fluralaner. This suggests that the treatments did not impact the feeding success of the insects (Figs. 2B–E, 3). The feeding success of *R. prolixus*, *T. infestans*, *T. brasiliensis* and *T. pseudomaculata* that fed on untreated chickens, chickens treated with a single dose or two doses of fluralaner, was similar (Fig. 2B–E).

### Fluralaner (Exzolt^®^) treatment of chickens induces insecticidal activity against triatomines

No side effects were observed in the chickens treated with a single dose or two doses of 0.5 mg/kg during the entire observation period (56 days after treatment). The assessment of insecticidal activity was based on the cumulative mortality of triatomines on day 7 after the blood meal. No mortality was observed in triatomine specimens (*R. prolixus, T. infestans, T. brasiliensis* and *T. pseudomaculata*) that fed on untreated control chickens.

**Fig. 4:**
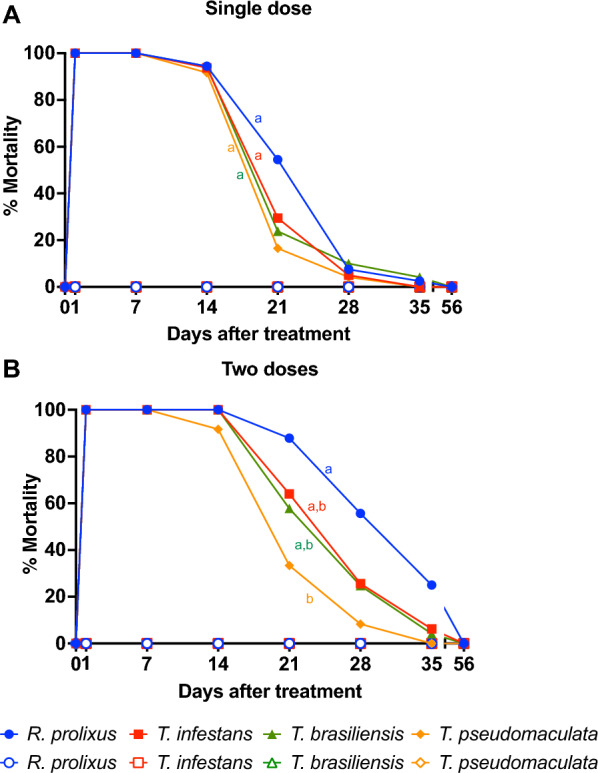
**A, B** Treatment with two doses of fluralaner (Exzolt^®^) induces higher insecticidal activity in *Rhodnius prolixus* compared to *Triatoma pseudomaculata* after a blood meal on treated chickens. Chickens were treated with one (**A**) or two (**B**) doses of 0.5 mg/kg fluralaner (Exzolt^®^), and mortality (%) of *R. prolixus, Triatoma infestans****,**** Triatoma brasiliensis and Triatoma pseudomaculata* was assessed before treatment and 1, 7, 14, 21, 28, 35 and 56 days after treatment. Ten nymphs of 3rd, 4th and 5th instars were fed on each chicken at each time point. Data are shown as means; a GLMM was conducted to compare mortality among groups. Mortality curves with different lowercase letters are significantly different at *P* < 0.05. Closed symbols represent insects that fed on fluralaner-treated chickens, open symbols represents insects that fed on untreated control chickens

Mortality was significantly higher in the fluralaner single-dose treated group up to 21 days after treatment for *R. prolixus* (GLMM, *F* = 70.57, *df* = 20.27, *P* < 0.001) and up to 14 days after treatment in *T. infestans* (GLMM, *F* = 176.58, *df* = 23.59, *P* < 0.001),* T. brasiliensis* (GLMM, *F* = 95.34, *df* = 20.17, *P* < 0.001) and *T. pseudomaculata* (GLMM, *F* = 83.68, *df* = 21.57, *P* < 0.001) compared to mortality (0%) before treatment (Fig. 4A; Table 1).

**Table 1:** Triatomine mortality after a single dose of fluralaner (0.5 mg/kg) was administered to chickens

Days after treatment	1	7	14	21	28	35	56
*Rhodnius prolixus* [% (*P*-value)]	100 (< 0.001)	100 (< 0.001)	94.4 (< 0.001)	54.4 (< 0.001)	7.5 (= 1.000)	2.5 (= 1.000)	0 (= 1.000)
*Triatoma infestans* [% (*P*-value)]	100 (< 0.001)	100 (< 0.001)	93.7 (< 0.001)	29.4 (= 1.000)	5.0 (= 0.428)	0 (= 1.000)	0 (= 1.000)
*Triatoma brasiliensis* [% (*P*-value)]	100 (< 0.001)	100 (< 0.001)	94.4 (< 0.001)	23.8 (= 0.062)	10 (= 1.000)	4.1 (= 1.000)	0 (= 1.000)
*Triatoma pseudomaculata* [% (*P*-value)]	100 (< 0.001)	100 (< 0.001)	91.6 (< 0.001)	16.5 (= 0.893)	4.1 (= 1.000)	0 (= 1.000)	0 (= 1.000)

Statistical analysis was performed using a generalized linear mixed model (GLMM) for repeated measures comparing triatomine mortality before treatment with that of the different periods evaluated after treatment

Mortality was significantly higher in the group treated with two doses of fluralaner up to 28 days after treatment in *R. prolixus* (generalized linear mixed model, GLMM, *F* = 70.57, *df* = 20.27, *P* < 0.001), *T. infestans* (GLMM, *F* = 176.58, *df* = 23.59, *P* < 0.001) and *T. brasiliensis* (GLMM, *F* = 95.34, *df* = 20.17, *P* < 0.001), and up to 21 days in *T. pseudomaculata* (GLMM, *F* = 83.68, *df* = 21.57, *P* < 0.001), compared to mortality (0%) before treatment (Fig. 4B; Table 2).

**Table 2:** Triatomine mortality after two doses of fluralaner (0.5 mg/kg) were administered to chickens

Days after treatment	1	7	14	21	28	35	56
*Rhodnius prolixus* [% (*P*-value)]	100 (< 0.001)	100 (< 0.001)	100 (< 0.001)	87.8 (< 0.001)	55.6 (< 0.001)	25 (= 0.520)	0 (= 1.000)
*Triatoma infestans* [% (*P*-value)]	100 (< 0.001)	100 (< 0.001)	100 (< 0.001)	64 (< 0.001)	25.6 (= 0.004)	6.2 (= 1.000)	0 (= 1.000)
*Triatoma brasiliensis* [% (*P*-value)]	100 (< 0.001)	100 (< 0.001)	100 (< 0.001)	57.7 (< 0.001)	24.7 (= 0.048)	4.1 (= 1.000)	0 (= 1.000)
*Triatoma pseudomaculata* [% (*P*-value)]	100 (< 0.001)	100 (< 0.001)	91.6 (< 0.001)	33.3 (= 0.040)	8.3 (= 1.000)	0 (= 1.000)	0 (= 1.000)

Statistical analysis was performed using GLMM for repeated measures comparing triatomine mortality before treatment with that of the different periods evaluated after treatment

**Fig. 5:**
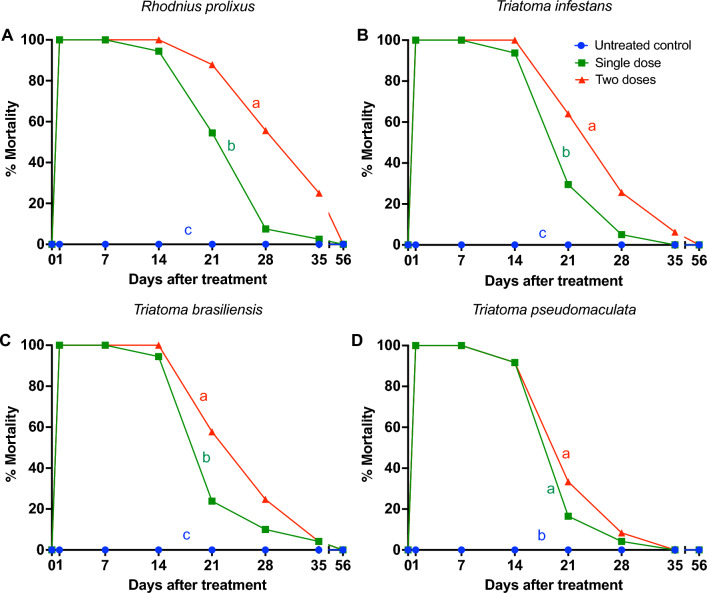
**A, B** Treatment with two doses of fluralaner (Exzolt^®^) showed higher insecticidal efficacy for *Rhodnius prolixus, Triatoma infestans* and *Triatoma brasiliensis* than a single-dose treatment. Chickens were treated with one or two doses of 0.5 mg/kg fluralaner (Exzolt^®^), and mortality (%) of *Rhodnius prolixus* (**A**),* Triatoma infestans* (**B**),* Triatoma brasiliensis* (**C**) *and Triatoma pseudomaculata* (**D**) was assessed before treatment and 1, 7, 14, 21, 28, 35 and 56 days after treatment. Ten nymphs of the 3rd, 4th and 5th instar were allowed to feed on each chicken at each time point. Data are shown as means, and a generalized linear mixed model (GLMM) was conducted to compared mortality among groups. Mortality curves with different lowercase letters are significantly different at *P* < 0.05. Closed symbols represent insects that fed on fluralaner-treated chickens, open symbols represent insects that fed on untreated control chickens

Taken together, our results demonstrated a higher variation and overall decline in mortality between the triatomines that fed on different chickens in the single-dose fluralaner treatment compared to the two-dose treatment (Fig. 5). In fact, treatment with two doses of fluralaner showed higher insecticidal efficacy for *R. prolixus* (*P* = 0.045) (Fig. 5A),* T. infestans* (*P* < 0.001) (Fig. 5B) and *T. brasiliensis* (*P* = 0.047) (Fig. 5C) compared to treatment with a single dose. Similar insecticidal efficacy was observed for *T. pseudomaculata* (*P* = 1.000) (Fig. 5D) using one or two doses of fluralaner. Treatment with two doses of fluralaner, when compared to a single dose, generated insecticidal activity for a longer period of time in *R. prolixus* (21 versus 28 days after treatment), *T. infestans* (14 versus 28 days after treatment), *T. brasiliensis* (14 versus 28 days after treatment) and *T. pseudomaculata* (14 versus 21 days after treatment) (Tables 1, 2). Moreover, mortality exhibited lower variation in the two-dose treatment compared to the single-dose treatment. The mortality curve was similar for all triatomine species (Fig. 5A). However, treatment with two doses of fluralaner generated higher insecticidal activity for *R. prolixus* compared to *T. pseudomaculata* (GLMM, *F* = 3.73, *df* = 27.80, *P* = 0.014) (Fig. 4B). These results indicate differences in susceptibility to fluralaner among the different triatomine species.

## Discussion

Treatment of chickens with fluralaner (Exzolt^®^) induced insecticidal activity against triatomines for up to 28 days post-treatment. To the best of our knowledge, this is the first study to evaluate the insecticidal activity of fluralaner (Exzolt^®^), a specific formulation for chickens, against triatomine species of epidemiological importance in the transmission of *T. cruzi* to humans in Latin America. Treatment of chickens with fluralaner (Exzolt^®^) appears to be a promising method for the control of Chagas disease by reducing the triatomine population, especially in the peridomestic environment.

Treatment of chickens with a single dose or two doses of fluralaner did not affect the feeding success and engorgement level of the triatomines compared with those that fed on untreated control chickens. Durden et al. [38] showed that treatment of chickens with fluralaner (Bravecto^®^) did not affect feeding success or engorgement level of *Triatoma gerstaeckeri*. Several studies have demonstrated that some insecticides, such as pyrethroids, have repellent effects against sand flies [40–42]. A repellent effect of the insecticide fluralaner administered to chickens could prevent the biting and blood-feeding of triatomines on the birds and induce a search for other hosts, such as other domestic reservoirs of Chagas disease and humans. However, as demonstrated here, treatment with fluralaner does not generate a repellent effect on *R. prolixus, T. infestans, T. brasiliensis* or *T. pseudomaculata*, indicating that it holds promise for use in control strategies.

Treatment with two doses of fluralaner showed higher insecticidal efficacy for *R. prolixus, T. infestans* and *T. brasiliensis* compared to the single-dose treatment. Moreover, there was high variation in the mortality of triatomines that took a blood meal from birds treated with a single dose. However, the insecticidal efficacy was similar for *T. pseudomaculata* in the single and two-dose treatments with fluralaner. The insecticidal activity of fluralaner (Exzolt^®^) against the triatomine bugs was observed for up to 21 and 28 days after treatment with one and two doses of fluralaner, respectively. There was no significant difference in the mortality of bed bugs (*Cimex lectularius*), which belong to a different family of insects than triatomines, that fed on chickens that received a single dose of 2.5 mg/kg or two doses of 0.5 mg/kg fluralaner (Bravecto^®^) [43]. That study [43] also reported higher variation in the mortality of the insects that took a blood meal from chickens treated with a single dose of fluralaner compared with two doses of fluralaner. However, a single dose of 2.5 mg/kg fluralaner (Bravecto^®^) was used against the bed bugs [43], whereas in the present study a single dose of 0.5 mg/kg fluralaner (Exzolt^®^) was used against the triatomines.

Treatment of the chickens with two doses of 0.5 mg/kg fluralaner (Exzolt^®^), the formulation and dosage recommended for chickens by the manufacturer, resulted in 100% mortality in *R. prolixus, T. infestans* and *T. brasiliensis* up to 14 days after treatment. *T. pseudomaculata* exhibited 100% mortality on days 1 and 7, and 91.6% mortality after 14 days of treatment. Triatomine mortality was observed up to 28 days after treating the chickens. A recent study [38] demonstrated that treating chickens with two doses of fluralaner (Bravecto^®^), a specific formulation for dogs, resulted in 100%, 90% and 50% mortality of *T. gerstaeckeri* at days 3, 7 and 14 after a blood meal, respectively. No mortality of *T. gerstaeckeri* was observed 28 and 56 days after treating the birds [38]. The higher insecticidal activity observed against *R. prolixus, T. infestans, T. brasiliensis* and *T. pseudomaculata* compared to that reported in the literature for *T. gerstaeckeri* might be explained by intrinsic resistance of the triatomine species to fluralaner and the drug formulation used. The insecticidal activity determined for triatomines is comparable to that observed for poultry red mites (*Dermanyssus gallinae*) using Exzolt^®^ [44], and for bed bugs (*C. lectularius*) using Bravecto chicken treatment [43]. Treatment of chickens with fluralaner (Exzolt^®^) resulted in 100% mortality of poultry red mites (*D. gallinae*) up to 15 days after treatment, and insecticidal activity against *D. gallinae* persisted up to 26 days after treatment [44]. Similarly, insecticidal activity of fluralaner (Bravecto^®^) was observed against *C. lectularius* up to 28 days after treatment of chickens [43]. It is worth noting that treatment of chickens with fluralaner (Bravecto^®^) may be less effective than their treatment with fluralaner (Exzolt^®^). Pharmacokinetic studies in chickens have demonstrated that the oral administration of two doses of 0.5 mg/kg fluralaner (Bravecto^®^) and fluralaner (Exzolt^®^) provides detectable levels of fluralaner in plasma for up to 14 and 21 days, respectively [38, 45, 46]. In vivo, fluralaner binds to plasma proteins and can accumulate in cells such as adipocytes, and in the skin, potentially prolonging its insecticidal activity [46, 47]. Therefore, a withdrawal period of 14 days after the last administration of Exzolt^®^ is recommended before meat and offal are consumed, although no withdrawal period is required for eggs [46].

There was variation in the insecticidal efficacy of fluralaner (Exzolt^®^) against the different species of triatomines. However, mortality was not affected by the engorgement level across different triatomine species. *R. prolixus* exhibited the highest success with respect to blood-feeding, followed by *T. infestans* and *T. brasiliensis*. Conversely, *T. pseudomaculata* displayed the lowest success with respect to blood-feeding and engorgement level. The amount, and rate, of blood ingestion during a blood meal in birds was highest for *T. infestans* (350 mg of blood ingested), followed by *T. brasiliensis* (286 mg) and *T. pseudomaculata* (75 mg) [48, 49]. The mortality of engorged bed bugs (*C. lectularius*) that fed on chickens treated with fluralaner (Bravecto^®^) was higher than that of partially engorged insects [43]. However, the mortality of fully engorged and partially engorged *T. gerstaeckeri* after a blood meal from chickens treated with fluralaner (Bravecto^®^) was similar. In contrast with mortality in bed bugs (*C. lectularius*) following ingestion of fluralaner [43], the mortality rate of *T. pseudomaculata* was high (91% for the single and two-dose treatments), although they were less successful in feeding. Outcomes might also be influenced by intrinsic differences between the species of insects used, the formulation of fluralaner (Bravecto^®^ and Exzolt^®^) used, e.g. the former is specific for dogs (chewable tablets) and the latter for birds (a solution administrated in drinking water), and the substantial variation in plasma concentrations of chickens treated with the same dose of fluralaner [38].

We observed a significant difference between the efficacy of a single dose and two doses of fluralaner (Exzolt^®^) at 0.5 mg/kg fluralaner (Exzolt®). The two-dose treatment showed prolonged insecticidal activity, lasting for up to 28 days after treatment of the birds. Moreover, fluralaner (Exzolt^®^) demonstrates low toxicity, allowing one to consider using higher therapeutic doses to extend insecticidal activity against triatomines [46]. In addition, the treatment of birds with two doses of 2.5 mg/kg fluralaner, administered 7 days apart, does not induce side effects [43, 50–52]. Thus, in intervention areas, fluralaner has potential as a treatment for chickens to reduce the triatomine population and, consequently, the number of domestic reservoirs and incidence of Chagas disease in humans.

Oral formulations of fluralaner have several advantages when compared to conventionally applied pyrethroids, which are currently used to control triatomines and hence Chagas disease. For example, the domestic application of pyrethroids requires the setting up of installations prior to spraying, for which the associated transportation and labor costs are high; resistance to pyrethroids has already been described for triatomines in different regions; the sprayed insecticide may not reach all of the locations in which the insects are present; and the residual effect of the insecticide in the peridomestic environment may be reduced (by 4–12 weeks) due to environmental conditions [53, 54]. On the other hand, fluralaner is simple to administer orally to animals, using palatable tablets for dogs or in drinking water for chickens; its insecticidal effect is independent of environmental conditions such as sunlight, rain and wind; no resistance to fluralaner has been described for triatomines; it has a favorable safety profile, and high specificity for the nervous system of insects; long residual efficacy has been observed. Treating dogs with fluralaner in locatios infested with *T. infestans* in the Chaco region of Argentina reduced the population of the insect, reduced the level of contact between triatomines and dogs and humans, and led to a reduction in the infection of triatomines with*T. cruzi* for 10–22 months after treatment, with effects recorded as early as 1 month after treatment [53]. Therefore, treatment with fluralaner is considered a promising strategy for use in Chagas disease control. Nevertheless, the cost-effectiveness of both formulations needs to be assessed [54, 55]. For informational purposes, a liter of fluralaner (Exzolt^®^) is sufficient for the complete treatment (two doses of 0.5 mg/kg) of 5000 chickens weighing 2 kg each, the cost of each liter is approximately US $1000 in April 2024 in Brazil. Thus, the treatment cost for each animal is approximately US $0.2.

Chickens and dogs serve as crucial sources of blood meals for triatomines in areas endemic for Chagas disease. Furthermore, dogs are an important domestic reservoir of *T. cruzi* in various Latin American countries [56–65], while chicken coops function as important shelters and breeding sites for triatomines [26–30]. Thus, interventions targeting chickens and dogs hold promise for the control of Chagas disease in humans. Fluralaner (Bravecto) administered to dogs demonstrated 100% insecticidal efficacy, lasting for 3–7 months post-treatment, against *T. infestans* [34, 35], *T. brasiliensis* [36], *R. prolixus* [37] and *T. gerstaeckeri* [66]. Furthermore, treatment of dogs with fluralaner (Bravecto) has been shown to reduce the level of infestation by *T. infestans* in sites in an endemic area in Argentina [53]. The novel control strategy proposed here, which includes treatment with fluralaner for chickens and dogs in association with existing treatments, such as pyrethroid insecticides, that have residual action in domestic and peridomestic environments, has potential for enhancing the efficacy of Chagas disease control.

## Conclusions

The findings presented here demonstrate that the treatment of chickens with fluralaner (Exzolt^®^) induces insecticidal activity against triatomines for up to 28 days post-treatment. All of the triatomine species studied showed susceptibility to fluralaner. Thus the treatment of chickens with fluralaner (Exzolt®) is a promising control strategy for Chagas disease in endemic areas.

## Data Availability

Data supporting the conclusions of the present study are included within the article.
